# Attributions, future time perspective and career maturity in nursing undergraduates: correlational study design

**DOI:** 10.1186/s12909-016-0552-1

**Published:** 2016-01-25

**Authors:** Cheng Cheng, Liu Yang, Yuxia Chen, Huijing Zou, Yonggang Su, Xiuzhen Fan

**Affiliations:** School of Nursing, Shandong University, 44 Wenhua Xi Road, Jinan, Shandong 250012 P. R. China; School of Foreign Languages and Literature, Shandong University, Jinan, Shandong P. R. China

**Keywords:** Career maturity, Attributions, Future time perspective, Nursing undergraduates

## Abstract

**Background:**

Career maturity is an important parameter as nursing undergraduates prepare for their future careers. However, little is known regarding the relationships between attributions, future time perspective and career maturity among nursing undergraduates. The purpose of this study was to investigate the degree of career maturity and its relationship with attributions and future time perspective.

**Methods:**

A cross-sectional survey was designed. This survey was administered to 431 Chinese nursing undergraduates. Independent-sample *t*-tests and one-way ANOVA were performed to examine the mean differences between categories of binary and categorical demographic characteristics, respectively. Pearson correlations and multiple linear regressions were used to test the relationships between attributions, future time perspective and career maturity.

**Results:**

The degree of career maturity was moderate among nursing undergraduates and that internal attributions of academic achievement, future efficacy and future purpose consciousness were positively associated with career maturity (all *p* < 0.01). These three factors accounted for 37.6 % of the variance in career maturity (adjusted R^2^ = 0.376).

**Conclusions:**

These findings might assist nursing educators and career counselors to improve nursing undergraduate career maturity by elucidating the imperative roles of internal attributions and future time perspective and to facilitate their transition from school to clinical practice.

## Background

Career maturity, as one of the main areas of career development guidance [1], is an essential factor for career preparedness in the twenty-first century [2]. The term refers, broadly, to the individual’s readiness to make informed, age-appropriate career decisions and to successfully manage appropriate career development tasks [3]. Meanwhile, as a primary aspect of career development, career maturity may predict individual occupational satisfaction and career success throughout one’s life [3, 4]. College students with higher career maturity exhibited a greater realization of their potential and a higher degree of social adjustment than those with lower career maturity [5]. Moreover, career maturity was also found to have a positive effect on job attainment among young people with low levels of educational attainment [6]. Additionally, career maturity was moderately correlated with work commitment among Australian high school students [7].

Nowadays, shortages, maldistribution, and imbalanced skill mix of health professionals are still common difficulties faced by China and other countries [8]. As the mismatch between expectancies and the reality of the practice of nursing, a portion of nursing graduates experience transition shock [9]. With respect to nursing students, improved career maturity combined with appropriate career strategies is important as nursing students move forward in their future profession [10]. Meanwhile, work readiness may facilitate a smooth transition into the workplace, may have a positive impact on job satisfaction and work engagement and may predict the intention to remain in the profession [11]. For nursing educators, assessing nursing undergraduate career maturity may prepare undergraduates with sufficient career knowledge and skills and facilitate their transition into clinical practice. Therefore, it is essential to identify the degree of career maturity and the associated factors affecting career maturity among nursing undergraduates.

### Career maturity

Career maturity as a construct of career psychology principally reflects an individual’s degree of mental maturity. According to the career maturity model [12], the construct includes both cognitive and affective dimensions. The cognitive dimension is represented by career decision-making competencies, while the affective dimension takes into account the individual’s attitude toward career development. Career maturity, defined as the individual’s ability to make appropriate career choices, comprises an awareness of what is required to make a career choice and the extent to which one’s career decisions are both realistic and consistent over time [13].

Studies on career maturity have received renewed attention in the last few years. K-N Kim and S-H Oh [14] found that social constrains due to low socioeconomic status had negative effects on career maturity among Korean students. Additionally, a longitudinal study found that career maturity developed continuously and positively over four years and that parental attachment was positively related to career maturity development among Korean adolescents [15]. In addition, secondary school students with high professional maturity were found to be aware of the importance of academic success for their future career goals, and thus, they were able to motivate themselves accordingly [16]. With regard to medical education, a previous study found that medical students had greater degree of career maturity in a traditional 4-year program during the early stages of a physician’s career than those in an accelerated program [17]. However, there is limited knowledge concerning career maturity among nursing undergraduates. Therefore, the need for more research to understand nursing undergraduate career maturity is of critical concern to educators.

### Attributions

According to attribution theory [18], people have some dominant causal perceptions regarding achievement-related contexts. The perceived causes of success and failure are directly hypothesized to influence people’s emotions, motivations and subsequent behaviors. B Weiner [19] classified all attributions into three properties – causality, which is either internal or external to the individual; stability, which is constant or varying over time; and controllability, which is within one’s volition to control or not. Accordingly, attributional style is regarded as the extent to which one tends to use the same combination of these causes over time [20]. According to Weiner’s theory [19], people who attribute career decision making to external factors (external attributions) believe that career-related events are caused by uncontrollable attributes, while those who attribute career decision-making to internal causes (internal attributions) believe that career-related outcomes are the result of determination and diligence [21].

Researchers have applied Weiner’s theory to explain individual career development. For example, IN Janeiro [22] found that positive career attitudes are associated with internal attributions for success among Portuguese students in grade 9 and grade 12, while GN Burns, D Jasinski, S Dunn and D Fletcher [23] posited that internal locus of control is positively correlated with career decision making self-efficacy among student athletes. For nursing students, a study found that nursing students’ causal attributions for success had significant influences on their self-regulated learning in pathophysiology course [24]. However, few studies focused on the relationship between attributions and career maturity among nursing undergraduates. Personal attributions are therefore worth considering when attempting to understand individual career maturity among nursing undergraduates.

### Future time perspective

Future time perspective, as a psychological factor, can predict an individual’s motivational goal-setting process for future career development [25]. Future time perspective, in particular, has been defined as an acquired mental characteristic that arises from future development goals [26] or as the present anticipation of future goals [27]. People with an extended or deep future time perspective set their motivational goals in the distant future and strive toward their goals by developing their present behaviors [28]. I Janeiro and J Marques [29] argued that being oriented toward the future is positively correlated with career maturity among secondary school students. Students who are more future oriented might be more determined in their study behaviors and may perceive their present studying to be more valuable and meaningful [30]. For health care professionals, a meta-analysis found that future time perspective as a predictor demonstrated a medium effect size on positive health practice [31]. As appreciated by previous educational and vocational research on the importance of future time perspective, it is necessary to investigate its relationship with career maturity among nursing undergraduates.

Based on the literature review, we assume that attributions and future time perspective may play important roles in nursing undergraduate career maturity. The purpose of the current study was to investigate the degree of career maturity and the relationships between attributions, future time perspective and career maturity among Chinese nursing undergraduates.

## Methods

### Design, participants and setting

This study was based on data from a survey of cross-sectional design. A convenience sample of 460 nursing undergraduates from two universities in Shandong Province, China participated in this study. Of the 460 participants, 431 with response rate of 93.7 % completed all of the questionnaires. All of the participants were full-time nursing undergraduates enrolled in their fourth or fifth academic year of five-year program. In China, nursing undergraduates attend a five-year program with a Bachelor degree in Medicine or a four-year program with a Bachelor degree in Science. Nursing undergraduates take theoretical courses in school during their first three or four years. In the final year, they start with their clinical internships in mainly hospital-based clinical settings. The current study enrolled nursing undergraduates who were in five-year nursing programs. A previous study found that students at the end of their Bachelor of Nursing program were more dissatisfied than the first and second year students with their preparation for work as nurses, which might be explained by that nursing undergraduates’ experience in the hospital settings make them better understand the gap between work preparation and the reality of the workplace [32]. As nursing undergraduates in the fourth and the fifth year are confronted with the challenge of clinical practice and role transition from students to nurses, it is of importance to pay close attention to their career preparation and development. Thus, the current study focused on undergraduates in the fourth and the fifth year of nursing programs. The questionnaire was administered to participants in their classrooms. Data were collected using hardcopy forms of the questionnaire, which took approximately 15 min to complete.

### Measures

#### Career maturity

The Chinese student career maturity inventory [33] was used to assess the nursing students’ level of career maturity. The inventory consisted of 34 items that assessed six factors - career decisiveness, career confidence, career independence, career value, relational dependence, and career reference. Career decisiveness referred to the extent of firmness about one’s preferred orientation toward career; career confidence reflected the degree of one’s faith and sureness of success in the chosen career; career independence referred to the degree of one’s independent career decision making [34]; career value indicated the career-related beliefs and ideas that are important to individuals and guide their career choice actions; relational dependence reflected the extent of one’s dependence on relatives’ and friends’ opinion; career reference indicated one referred to experts’ intention or behaviors when they chose their career. Example items were shown in Table 1. Participants were asked to complete the self-reported career maturity inventory based on their perceptions of each item. Responses were rated using a 5-point Likert scale (1 = completely disagree to 5 = completely agree). The total mean score was computed by averaging of the summing of the items, with higher total scores indicating higher degrees of career maturity. The inventory had a reasonable internal consistency coefficient with Cronbach’s alpha = 0.869 [33]. The Cronbach’s alpha for this sample was 0.76.

**Table 1:** Construct subscales and sample items

	Subscale	Sample items
Career maturity inventory	Career decisiveness	I have made a decision to engage in what kind of work in the future.
	Career confidence	I was worried that I could not find an expected job.
	Career independence	No matter what others say, I think I will choose the career I prefer to.
	Career value	The most important factor I would consider in career choice is money.
	Relational dependence	I would make a career choice in accordance with my parents’ expectation.
	Career reference	I want to communicate with people in my interested occupational field.
Multidimensional-multiattributional causality scale	Attributions of academic achievement	Ability: The most important factor for getting good grades is my ability of learning.
		Effort: I attributed my good grades to efforts.
		Contextual factors: Sometimes I got a good grade just because of the easy contents of the course.
		Luck: Sometimes I have to rely on luck to get the success of examination.
	Attributions of affiliation	Ability: Getting along with people is a skill.
		Effort: Efforts are needed to maintain friendship.
		Contextual factors: Whether I enjoy a social occasion almost completely depends on the characteristics of others.
		Luck: Making friends is mostly a matter of taking my chance.
General future time perspective questionnaire	Behavioral commitment	I am gradually moving forward to finish my plan on time.
	Future efficacy	I am confident of my future.
	Far-reach goal orientation	I often imagine the goal I am to achieve in five years.
	Future purpose consciousness	I often feel living without an aim.
	Future image	I know there are a lot of tasks to be done in the future.

#### Attributions

The multidimensional-multiattributional causality scale (MMCS) developed by HM Lefcourt, CL Von Baeyer, EE Ware and DJ Cox [35] was used to investigate different attributional styles among nursing undergraduates. The Chinese version of MMCS was adopted in the current study, which has been widely used in Chinese mental health assessments [36]. The scale was comprised of two subscales for affiliation and academic achievement. Each subscale, consisting of 24 items, contained four dimensions: ability, effort, contextual factors and luck. Example items were shown in Table 1. Items were rated from 0 (strongly disagree) to 4 (strongly agree). Scores of internal attributions were calculated by adding scores of ability and effort together, while scores of external attributions were the sum of scores for contextual factors and luck. The total score was then calculated by subtracting the total internality score from the total externality score. Ranging from −48 to 48, the total score provided a general indication about the propensity for attributions. Namely, scores from −48 to 0 indicated the degree of internal attributions, while a score from 0 to 48 reflected the degree of external attributions. A higher absolute value indicated a higher degree of internal (or external) attributions. The Cronbach’s alpha for MMCS in a Chinese sample was 0.79 [37], and in this study, the Cronbach’s alpha was 0.85.

#### Future time perspective

The general future time perspective questionnaire [38] was used to examine nursing undergraduates’ future time perspective. This 20-item inventory measured five factors - behavioral commitment, future efficacy, far-reach goal orientation, future purpose consciousness and future image - of future time perspective among university students. Specifically, behavioral commitment reflected an individual’s tendency to put established goals into action; future efficacy indicated an individual’s confidence about the future; far-reach goal orientation reflected an individual’s concern regarding long-term life goals; future purpose consciousness assessed an individual’s clear cognition regarding the future; and future image reflected an individual’s image of events or tasks in the near and distant future. Example items were shown in Table 1. Respondents were asked to indicate on a 4-point scale ranging from 1 (strongly disagree) to 4 (strongly agree) the extent to which they agreed with each of several statements. Higher scores indicated higher levels of future time perspective. The total mean score of future time perspective ranged from 1 to 4. QZ Song [38] conducted a factor analytic study and reported a test-retest reliability coefficient of 0.79 and Cronbach’s alpha of 0.90. For the current sample, Cronbach’s alpha was 0.83.

#### Socio-demographics

Based on the literature on career maturity [39], this questionnaire gathered an array of demographic data, which included age, gender (1 = male, 2 = female), study phase (1 = fourth year and 2 = fifth year) and social practice experiences (1 = no experience, 2 = experience not related to nursing profession, 3 = experience related to nursing profession). Social practice experiences refer to activities in which students participate during their spare time, such as volunteer service, charity activities, social surveys, part-time jobs, clinical novitiate or professional internships [40].

### Ethical considerations

Ethical approval was granted by the Medical Ethics Committee of Shandong University for all participants. Participants were given oral and written information prior to the study, including information on the purpose of the study, an assurance of confidentiality, and the right to withdraw from the study. Informed consent was obtained from all participants.

### Data analysis

Statistical analysis was performed using SPSS 19.0. Descriptive statistics such as means, standard deviations and percentage distributions were used to organize the data. For career maturity scores, the Kolmogorov-Smirnov test was conducted to determine the normality of the distribution. Because the career maturity scores showed normal distribution (*p* > 0.05), independent-sample *t*-tests and one-way ANOVA were performed to examine the mean differences between categories of binary and categorical demographic characteristics, respectively. Pearson correlations were used to test the relationships between attributions, future time perspective and career maturity. Multiple linear regressions were performed to identify the association of career maturity with attributions and future time perspective. The statistical significance level was set to *p* < 0.05.

## Results

### Socio-demographic characteristics

As evidenced in Table 2, of the 431 participants in this study, the majority were female (*n* = 394, 91.4 %), and they ranged in age from 19 to 25 (M = 22.53, SD = 0.92). There were 149 (34.6 %) fifth year students and 282 (65.4 %) fourth year students. The proportion of nursing undergraduates with social practice experiences was 91.9 % (*n* = 396).

**Table 2:** Demographic characteristics and career maturity score differences by demographic characteristics (*N* = 431)

Variables	n (%)	Career maturity		t/F value	*p*
		Mean	SD		
Gender					
Female	394 (91.4)	3.23	0.32	−1.68	0.094
Male	37 (8.6)	3.14	0.37		
Study phase					
Fourth year	282 (65.4)	3.22	0.33	−0.51	0.608
Fifth year	149 (34.6)	3.24	0.31		
Social practice experiences					
Yes, related to the profession	247 (57.3)	3.23	0.32	1.45	0.237
Yes, not related to the profession	149 (34.6)	3.23	0.33		
No	35 (8.1)	3.14	0.33		

### Career maturity and its differences among groups

The mean score for career maturity among the 431 sampled students was 3.23 (SD = 0.33). With respect to the sub-dimensions, the mean values (ranging from 1 to 5) varied between 3.00 and 3.51. More specifically, the mean values were as follows: career decisiveness 3.11 (SD = 0.61), career confidence 3.00 (SD = 0.60), career value 3.27 (SD = 0.61), career independence 3.51 (SD = 0.56), relational dependence 3.33 (SD = 0.59), and career reference 3.30 (SD = 0.42). Ratings of career independence were the highest, while those for career confidence were the lowest.

As presented in Table 2, the comparison of mean score for career maturity between male and female students showed that female students had higher score than male students, but the difference was not significant. In addition, there were no significant differences in career maturity scores by study phases or social practice experiences.

### Correlations between career maturity and other variables

As presented in Table 3, correlations between career maturity and other variables were statistically significant and in the expected direction (*p* < 0.01). The mean scores for attributions in academic achievement and affiliation were negative, thus indicating the degree of internal attributions. The scores for internal attributions and future time perspective were positively correlated with score of career maturity, suggesting that higher internal attributions and higher future time perspective were correlated with greater career maturity. Some of the relationships between subscales of career maturity and other variables were also significant. Specifically, the internal attributions of academic achievement were significantly associated with subscales for career maturity, with the exception of career decisiveness, while the internal attributions of affiliation were solely significantly related to career decisiveness (*p* < 0.05). Negative values for correlation coefficients between career maturity scores and attribution variables indicated that career maturity increased with higher internal attribution scores. For subscales of future time perspective, behavioral commitment was positively correlated with subscales for career maturity, with the exception of the relational dependence. Finally, far-reach goal orientation was not significantly related to career value or relational dependence.

**Table 3:** Correlation coefficients among career maturity and various dimensions of attributions and future time perspective (*N* = 431)

Variables	Mean	SD	1	2	3	4	5	6	7
1. Career maturity	3.23	0.33							
2. CM–Career decisiveness	3.11	0.61	0.69**						
3. CM–Career confidence	3.00	0.59	0.73**	0.40**					
4. CM–Career value	3.27	0.61	0.55**	0.11*	0.34**				
5. CM–Career independence	3.51	0.56	0.52**	0.27**	0.20**	0.04			
6. CM–Relational dependence	3.33	0.59	0.53**	0.13**	0.40**	0.33**	0.13**		
7. CM–Career reference	3.30	0.42	0.28**	0.01	−0.02	−0.08	0.38**	−0.02	
8. Attributions of academic achievement	−4.86	5.86	−0.32**	−0.08	−0.23**	−0.24**	−0.22**	−0.24**	−0.19**
9. Attributions of affiliation	−1.84	4.93	−0.13**	−0.12*	−0.08	−0.05	−0.07	−0.08	−0.02
10. Future time perspective	2.74	0.36	0.54**	0.31**	0.45**	0.26**	0.36**	0.19**	0.28**
11. FTP–Behavioral commitment	2.51	0.49	0.31**	0.19**	0.26**	0.13**	0.25**	0.05	0.16**
12. FTP–Future efficacy	2.79	0.58	0.40**	0.25**	0.36**	0.19**	0.24**	0.10*	0.18**
13. FTP–Far-reach goal orientation	2.54	0.50	0.29**	0.24**	0.23**	0.03	0.28**	−0.01	0.21**
14. FTP–Future purpose consciousness	2.93	0.56	0.49**	0.24**	0.45**	0.31**	0.23**	0.31**	0.15**
15. FTP–Future image	2.99	0.49	0.39**	0.13**	0.27**	0.27**	0.25**	0.21**	0.29**

*Note: CM* career maturity, *FTP* future time perspective

*p < 0.05, **p < 0.01

The negative value of mean scores of attributions indicate the degree of internal attributions. Thus, the negative correlation coefficients reflect the positive relation between internal attributions and career maturity

### Regression analysis of career maturity

The multiple linear regression results were presented in Table 4, with the overall career maturity score as the dependent variable. Socio-demographic variables, subscales of attributions and future time perspective, as a set of independent variables, were entered into the regression model. After the multiple adjustments, three variables - internal attributions of academic achievement, future efficacy and future purpose consciousness - were significantly associated with higher score of career maturity and they accounted for 37.6 % of the variance in career maturity (R_adj_^2^ = 0.376).

**Table 4:** Multiple linear regression for career maturity (*N* = 431)

Variables	B	SE	β	t	95 % CI		Collinearity Statistics	
					lower	upper	Tolerance	VIF
Constant	1.920	0.347	—	5.538**	1.238	2.601	—	—
Attributions score of academic achievement	−0.009	0.002	−0.163	−3.744**	−0.014	−0.004	0.768	1.302
FTP–future efficacy	0.078	0.029	0.138	2.711**	0.021	0.135	0.562	1.780
FTP–future purpose consciousness	0.220	0.024	0.375	9.190**	0.173	0.267	0.871	1.149

*Note:* **p < 0.01

Dependent variable: career maturity score

F = 24.557, *p* < 0.01, R = 0.626, R^2^ = 0.392, R_adj_^2^ = 0.376, SE = 0.259

*VIF* variance inflation factor, *FTP* future time perspective, *95%CI* 95 % confidence interval

As negative value of attributions score presented the degree of internal attributions, the negative regression coefficient reflected the positive relationship between internal attributions of academic and career maturity

## Discussion

In this study, we investigated the level of career maturity and its relationship with attributions and future time perspective in Chinese nursing undergraduates. The results indicate that the degree of career maturity among Chinese nursing undergraduates was relatively high compared, for example, with that of other young people in Hong Kong, China [6]. While career maturity was higher among females than males, there was no significant difference in career maturity between the two genders in our study. This is consistent with the finding regarding career maturity among adolescents in Southwest Nigeria [41]. However, some studies found that females had a higher degree of career maturity than males [42–44]. This finding may be because the small number of male nursing undergraduates was not sufficient to demonstrate a statistically significant difference. Similarly, in the current study, it was found that there was no difference in career maturity based on age, a finding that conflicts with the findings of a previous study [39], but it is supported by another study that found that chronological age did not determine the degree of career aspiration development in adolescents [45].

As expected, internal attributions of academic achievement were positively associated with nursing undergraduates’ career maturity. This result is similar to the findings in a previous study that reported that internal attribution beliefs were positively related to career attitudes among adolescents [22]. It is possible that internal attributions might result in a change in self-efficacy, and thus have an impact on the individual’s behaviors. As SH Lease and DT Dahlbeck [46] reported, internal locus of control attributions were important in predicting career decision self-efficacy among males. Moreover, a study among Australian high school students found that internal attributions can influence career planning and career exploration [47]. Similarly, a longitudinal study illustrated that attributions could predict subsequent motivation of academic engagement among African American adolescents [48]. Attribution retraining was found to provide opportunities for improving the undergraduate nursing students’ vocational knowledge, professional knowledge, and social abilities [49]. Thus, with regard to nursing educators and career counselors, strengthening nursing undergraduates’ attribution training may facilitate the improvement of their career maturity. For instance, nursing educators and career counselors may induce undergraduates to explain their failures with appropriate internal attributions and encourage them to overcome difficulties through efforts [50]. Interventions for attributions retraining are likely to be helpful for nursing undergraduates to successfully master adequate nursing knowledge and skills, and to be matured and prepared for their future career.

This study found that future time perspective was positively related to career maturity. This finding is similar to a study that showed future time perspective was related to career decision-making among American university students [51]. Moreover, a recent study also found that future time perspective, as an important theoretical construct, can indirectly impact an individual’s commitment to career choices [52]. Additionally, college students with higher future time perspective were found to experience less employment pressure and take more positive coping styles [53]. Appropriate interventions with respect to time perspective were also found to improve adolescents’ ability to project into the future and foster their commitment to their decisions [54]. Accordingly, findings from the current study might assist nursing educators and career counselors in better understanding nursing undergraduates’ future time perspective, thus helping educators and counselors enhance nursing undergraduate future efficacy and future purpose consciousness intended to improve students’ career maturity. Interventions that foster nursing undergraduate future time perspective, such as career planning courses might be an effective strategy for nursing educators to encourage nursing undergraduates to look into the future, and determine their goals of career development.

When interpreting the current results, several limitations must be considered. First, given the cross-sectional survey design, the associations observed in this study cannot be inferred as causal relationships. Thus, a longitudinal study is necessary for further explanation of career maturity. Second, the findings are based on a sample of nursing undergraduates from two universities in China and are limited to be generalizable to other geographical areas. Third, students included were those in the 4th and 5th year of nursing program, which are considered the particularity of the two groups in the transition to clinical practice. Future studies should take consideration of students at different stages of a nursing program so as to help nursing educators provide career guidance in the early of nursing education.

## Conclusions

The degree of career maturity among Chinese nursing undergraduates is moderate in the present study. With respect to the relationship between attributions, future time perspective and career maturity, the present study suggests that internal attributions of academic achievement, future time perspective contribute to career maturity among Chinese nursing undergraduates. Thus, nursing educators and career counselors should pay more attention to augmenting career maturity among nursing undergraduates by strengthening their internal attributions and future time perspective. In addition, further research is needed to identify appropriate educational interventions to improve career maturity among nursing undergraduates.

## Abbreviations

CM: Career Maturity
MMCS: Multidimensional-Multiattributional Causality Scale
FTP: Future Time Perspective
